# Resilience beyond reductionism: ethical and social dimensions of an emerging concept in the neurosciences

**DOI:** 10.1007/s11019-020-09981-0

**Published:** 2020-10-12

**Authors:** Nikolai Münch, Hamideh Mahdiani, Klaus Lieb, Norbert W. Paul

**Affiliations:** 1 Institute for History, Theory and Ethics of Medicine, University Medical Center, Am Pulverturm 13, 55131 Mainz, Germany; 2 Department of Psychiatry and Psychotherapy, University Medical Center, Mainz, Germany

**Keywords:** Resilience, Enhancement, Ethics, Neuroethics, Bioethics

## Abstract

Since a number of years, popular and scientific interest in resilience is rapidly increasing. More recently, also neuroscientific research in resilience and the associated neurobiological findings is gaining more attention. Some of these neuroscientific findings might open up new measures to foster personal resilience, ranging from magnetic stimulation to pharmaceutical interventions and awareness-based techniques. Therefore, bioethics should also take a closer look at resilience and resilience research, which are today philosophically under-theorized. In this paper, we analyze different conceptualizations of resilience and argue that especially one-sided understandings of resilience which dismiss social and cultural contexts of personal resilience do pose social and ethical problems. On a social level such unbalanced views on resilience could hide and thereby stabilize structural social injustices, and on an individual level it might even lead to an aggravation of stress-related mental health problems by overexerting the individual. Furthermore, some forms of fostering resilience could be a latent form of human enhancement and trigger similar criticisms.

## Introduction

Since a number of years, scientific interest in human resilience is rapidly increasing especially in psychology (for a review see e.g., Fletcher and Sarkar 2013) and even more recently, with a higher appearance, in neurological research (e.g., Chmitorz et al. 2018; Feder et al. 2011, 2019; Kalisch et al. 2015, 2017; Osorio et al. 2017; Russo et al. 2012). Neurosciences have been and still are of major interest to philosophy and ethics and have even brought about the novel field of neuroethics. However, resilience research as well as resilience interventions which aim to promote or foster human resilience have surprisingly attracted little attention so far in philosophy, in general, and in ethics, in particular. This is despite the fact that concepts of resilience are regularly discussed in the light of liminal experiences and fundamental challenges in human life. Due to the lack of philosophical and ethical discourse and deliberation regarding resilience research and application, “the notion of resilience has to date been a philosophically under-theorized concept” (Lotz 2016, p. 49), with a few exceptions. For example, Russell (2015) has focused on sport philosophy. Elsewhere, Titus (2011) and Richter (2017) have studied resilience in relation to theological perspectives. This abstinence of ethics and philosophy in resilience research is quite lamentable given the fact that resilience as a concept as well as resilience interventions based on neurobiological findings undoubtedly pose philosophical, social, and ethical questions. While we neither argue for or against enhancement nor do we re-interpret resilience research and interventions by subsuming them as novel approaches to psychological and/or neuro-enhancement studies, we encourage those who see social or ethical problems with enhancement technologies to take a closer look at resilience and the related neurobiological concepts. In this paper, we argue that particular notions of resilience are crossing the sometimes fine line between maintaining a person’s mental health, despite the impact of severe psychological or physical adverse events (Kalisch et al. 2015, p. 1), and ethically more debatable discourses of enhancement. To do so, we will proceed in three steps.

1. In our first step, we describe the concept of resilience in general, and its neurobiological study in particular.
2. We attempt to show that a one-sided concept of resilience—as it is often presented in neurobiological research on resilience—does indeed pose social and ethical problems on three interrelated levels:
  (2.1) Fueled by neurobiological exceptionalism, resilience has the tendency to be an expanding discourse which might absorb debates on the question as to how the natural (environment), social (work, income, health), and cultural (protection of justified interests, education, meaningful life) spheres could be better adapted to human needs and pre-conditions to form strategies which would vest humans with abilities or traits to cope with the given situation. This is how, we argue, complementary perspectives on fundamental challenges in the lives of humans could be cut out.
  (2.2) Resilience has an implicit tendency toward normalization by applying normative standards (for example, social desirability) which are not explicitly stated and are rather hidden in value-ladden terms like ‘functioning.’ Such terms are in turn used in a teleological manner to define what ‘mental health’ or ‘resilience’ should be.
  (2.3) If neurobiological research on resilience is performed with a reductionistic and primarily overly individualistic conceptualization of resilience, it may lead to blind spots on the individual and social levels. Such an approach may lead to an aggravation of stress-related mental health problems and the related social-structural difficulties over a longer period of time.
3. In the last and final step of this paper, we argue that a one-sided reading of resilience can be understood as a latent form of enhancement in transition and as such it poses ethical questions similar to those discussed in bioethics in relation to other approaches to the biomedical enhancement of humans.

## Resilience research and the neurobiological study of resilience

Although it is a common belief among resilience researchers that many “people do not become, or only temporarily become, mentally ill despite significant or physiological burdens” (Kalisch et al. 2015, p. 2), there are wide discrepancies in the way resilience is defined and conceptualized. However, in almost all discussion of resilience, one comes across the two concepts of adversity and positive adaptation. These discrepancies in the concept itself are a reflection of the historical development of resilience research. If we want to summarise the resilience research conducted during the last decades, the simplest way might be, as is time and again referred to by other researchers, categorizing it into different interrelated waves. In this vein, a resilience researcher advocating to the first wave of resilience research defines resilience as a quality, termed protective factors or resources, within individuals (see e.g., Luthar and Chicchetti 2000; Masten 2001, 2007; Richardson 2002). Researchers more in line with the second wave go a step further and claim: it is the adaptive processes, or coping, that facilitate resilience (see e.g., Masten 2007; Ong et al. 2009; Richardson 2002) and therefore focus on the significance of environmental factors including the interactions between individuals and their environment (Wild et al. 2013). These two groups of researchers result in the concept of resilience being defined as either a trait or a process. One should also mention the other two waves of research which on the one hand try to foster resilience through interventions such as the promotion of positive parenting as advocated by Goldstein and Brooks (2005); and on the other hand, discuss biological and molecular traits and processes on genomic and neurobehavioral levels together with statistically valid associations of phenotypes to come up with a better understanding of the complex processes that lead to resilience (Masten 2007). Particularly approaches from a molecular perspective are good candidates for leading to new kinds of resilience interventions based on neurobiological mechanisms and so adding to merely behavioural resilience interventions (awareness-based practices like yoga or meditation). The most likely pathway to molecular interventions is the creation of pharmaceutical tools (King 2016; Russo et al. 2012).

In this context, conceptual and explanatory discrepancies primarily (but not only) stem from the following questions: Is resilience understood as a trait, an outcome or a process? What is the scope of adversity underlying resilience as a concept? Does resilience refer only to significant psychological or physical burdens (for example, war, rape etc. which statistically often cause posttraumatic stress disorders) or also to everyday stressors related to the daily challenges in one’s personal and professional life (see Davis et al. 2009) or something in-between? Furthermore, the extent to which resilience is regarded to be an individualistic or relational phenomenon is often—at least—ambiguous and demands further explication. In some definitions of resilience, familial, interpersonal or societal factors are—besides personal individualistic factors (character traits, genetics, etc.)—stressed, while other definitions point out that relational factors may only have a distant impact. While, for example, Luthar (2006, p. 780) declares “resilience rests, fundamentally, on relationships. The desire to belong is a basic human need and positive connections with others lie at the very core of psychological development,” Kalisch et al. (2015, p. 21) make it a strong point that they consider “socio-environmental factors on mental health […] as distant influences.”

With regards to these differences in defining the concept of resilience itself we acknowledge that resilience is (a) more or less dynamic and something one can “learn” (against something one is born with); (b) more or less tied to the prevention of major diseases (against something which is necessarily omnipresent to get along with in one’s everyday life), and finally (c) more or less an individual task or challenge (against a common or social task or challenge). These differentiations in the understanding of resilience create a strong undertow in neuroscientific research on resilience. The primarily neurobiological approaches (Kalisch et al. 2015; Russo et al. 2012) understand resilience as an outcome, which means that resilience can be described only “post factum” because it is inextricably tied to a prior adversity. While some stress the genetic and thereby heritable factors (for example, genes related to the regulation of the hypothalamus–pituitary–adrenal axis; see for example, Feder et al. 2009) others focus more on active and reactive neurobiological resilience mechanisms (for example, Kalisch et al. 2015). Furthermore, adversity in neuroscientific resilience research is sometimes understood in “the broadest sense” (Kalisch et al. 2015, p. 5) and includes “mildly aversive situations” (p. 15). However, others understand adversities as statistically and significantly related to major diseases as post-traumatic stress disorder (PTSD) and major depressive disorder (MDD) (for example, Russo et al. 2012). Notwithstanding, the focus of current neuroscientific research on resilience clearly lies on individuals’ active adaptation mechanisms and their biological basis and neurocognitive processes.

Having located the neurobiological perspectives within the field of resilience research, in what follows, especially in “Reductionist understandings of resilience” section, we argue that these new approaches should not be at the cost of a limited or absent attention to the socio-environmental factors (interpersonal relations, culture, etc.) of resilience, for this choice would come with ethical consequences.

## The social and ethical challenges of (neurobiological) resilience

In this section we address some of the challenges brought about by recent, particularly neurobiological and to some degree reductionist views of resilience research. However, we will not argue against the meaningfulness of the concept of resilience and resilience research per se. It must be stressed that—despite our critical remarks—we do not doubt that resilience research leads to insights that can be vested to improve the lives of people, neither do we claim that neurobiological resilience research is per se reductionistic or ethically problematic. Instead, we want to point to some potential pitfalls that could lead to one-sided understandings of resilience and resilience research. Therefore, we want to stress the point that neurobiological resilience research needs to be embedded in a broader understanding of resilience that incorporates (neuro-)biological as well as psychological and social aspects.

Against this background, we argue that resilience and resilience research yield social and ethical questions if performed in a one-sided manner. These questions and challenges arise on three different levels. Firstly, if resilience research is embedded in an undifferentiated and/or unspecific discourse with a tendency to encompass more and more aspects of daily life, it will thereby eliminate alternative perspectives and approaches to questions of mental and social well-being. This is a well-known effect as the expansion of explanatory models and the so-called bacteriological or genomic ages represent past expansions of per se meaningful but at times overinflated biomedical models. One danger of the expansion of the concept of resilience lies in its powerful behavioral components and its closeness to the concepts of social competition. This might cause an even more adverse reaction than the so called geneticization (Paul 2002) of human life (see “Resilience as an ever-expanding discourse” section), leading to the idea of exceptionalism which in turn has been more or less completely deconstructed by not only but mainly by ethical and social debates. Secondly, concepts of resilience are susceptible to be tacitly leading to the processes of normalization by defining and applying (seemingly) objective criteria for the desirability of resilience (from performance measures to neurobiological traits) and by interpreting deviations from them as indicators for a need—or at least a well-founded reason—to foster health in order to improve performance (see “Resilience and normalization” section). Finally, a major social and ethical challenge will be created if resilience research eliminates social perspectives on conditions leading to mental health problems by delegating the responsibility for resilience exclusively to affected individuals; this tendency is further reinforced by concentrating on individual neurobiological mechanisms (see “Reductionist understandings of resilience” section) in terms of individualized or precised medicine. These three challenges are not necessarily tied to neurobiological resilience research only. Also non-neurobiological resilience research faces these ethical challenges. But the recent wave of neurobiological research on resilience could foster these three tendencies, especially the latter one of understanding resilience as overly individualistic.

### Resilience as an ever-expanding discourse

Resilience is located in the discourse of prevention. Kalisch et al. (2017) speak of a “prevention gap” in the research investigating the pathophysiology of stress-related disorders that could and should be closed by (neurobiological) resilience research. The turn of this argument is interesting indeed, because resilience does not focus (like prevention) on diseases rather it focuses on fostering factors that maintain mental health. Despite this difference, prevention (of disease) and fostering resilience share some common features that tacitly lead to ever expanding discourses on prevention and resilience respectively (for a critique of preventive discourses in this respect see for example, Bröckling 2017a). On the one hand, in resilience research, risk-factors and positive adaptation mechanisms are understood as context-specific (see for example, Fooken 2016). Furthermore, concrete measures to strengthen resilience are generally quite unspecific (Bröckling 2017b, pp. 124–125). This is further aggravated by the fact that resilience is increasingly understood as basic “life skills,” which include very general capabilities such as “decision making, problem solving, creative thinking, and self-awareness” (WHO 1997, p. 1). In this sense, almost every social and individual measure to improve human life can be understood as a step to improve resilience. We therefore believe that concepts of resilience have the tendency to become rather a general understanding of life and reality without concrete instructions how to improve resilience. This includes a highly unspecific portfolio of practices reaching from prayer, meditation, yoga, and awareness-based training, on one side of the scale, to the application of pulsing magnet fields and brain stimulation, on the other.

From a more epistemological point of view, this creates yet another challenge. Whenever resilience is effective, potential risk-factors should not lead to negative psycho-cognitive impact or even mental disorders. However, the risk factors are still potentially present. Despite the declared focus on health, there seems to be no positive objective in the form of a positive definition of health (see e.g., Kalisch et al. 2015, p. 5) for example as a capability based approach focusing on the ability of individuals to lead their lives (social participation). This way, health comes into focus only as absence of disease. It becomes some unspecific, unreachable vanishing point leading to a configuration in which fostering resilience is some all-encompassing form of understanding reality and an indispensable pre-condition of successful life.

### Resilience and normalization

While it seems to be unfair to imply that resilience research is hinging on a utopian and under-explicated notion of health, one might contend that there are some objective criteria for resiliency beyond the ideal of health. These criteria can be found in concepts like “functioning,” academic achievement, social behavior in conformity with the prevailing rules, etc. (as in Masten et al. 2009, p. 119f). Evidently, those criteria are impregnated by implicit norms and ideas of normality. Particularly with regard to social behavior and in conformity with the prevailing rules and expectations, this seems quite obvious: to be resilient in the sense of Masten and her colleagues is to fit into the prevailing social order. However, “functioning” is difficult to define without implicating normative ideas. Tellingly, the term “functioning” remains undefined in both dimensions, the neurobiological and the behavioral. No doubt, it is difficult to define “functioning” in a neutral, non-value-laden way. The long history of attempts to define health in terms of “normal functioning” is an incessant struggle to find a definition above biological reductionism and beyond sheer normativity. An accepted neutral definition of health is not commonly agreed upon, a fact that holds particularly true if it comes to definitions of mental health for example Boorse (1976); for a critique see for example Lenk (2002, p. 126f). If no objective, neutral teleological criteria for resilience (here understood as the maintenance or improvement of mental health) is agreed upon, then the expansion of resilience as an explanatory concept is fueled by the tendency to view everybody as non-resilient who do not fit into existing (social) norms. In this regard, measures to strengthen resilience might implicitly foster processes of normalization in so far as they aim at an adaptation of individuals to given, pre-existing circumstances. This potential tacit normativity of resilience grows especially problematic in combination with the quite unspecific definitions and measures to foster resilience (see “Resilience as an ever-expanding discourse” section). put differently, such a mixture opens up the possibility that every research orexplanatory model and even many sociocultural practices (like reading novels or practicing prayer) can implicitly link resilience with some teleological dimension of what they think is valuable or desirable (see for example, Kaplan 1999). To make things even worse, it must be stressed that research on marginalized social groups has shown that nonconformist and in this sense seemingly “maladaptive” or “deficient” behavior can in turn reflect psychological strength and thus be interpreted as functional resilience (see Hahn 2011; Liebel 2011). In this regard, a well-defined notion of health related to resilience is highly in demand.

### Reductionist understandings of resilience

One response to this rather pessimistic analysis of the implicit tendency to normalization of concepts of resilience (that is not to prevent negative events, but to contain or neutralize their consequences) can be seen in a ubiquitous discourse of empowerment. Understanding resilience as a kind of personal empowerment is made possible by delegating the responsibility to foresee and prevent negative psycho-cognitive impacts of lived experience to the individual herself. If resilience is something one can learn and train, then everybody can do something to survive (or even grow) in the face of adversity. This view gets especially problematic if resilience as a discourse is focused solely on the individual and is not complemented by considering social problems and dysfunctional social structures. The tendency toward individualization seems to be strengthened by recent neurobiological research on resilience since in this context primarily the neurobiological mechanisms of individual resilience processes come into sight. This involves the danger that more encompassing socio-environmental factors get out of sight. This sided individualistic notion of resilience leads to blind spots regarding the social (a) as well as the individual level (b):

- If resilience, exclusively, aims at the individual ability to withstand stress, it ignores all the social circumstances which structurally cause mental burdens for the individuals involved. Therefore, resilience is criticized for fostering the depoliticization of individuals (for example, Neocleous 2013). We argue that structural social problems may be overlooked if every individual herself is responsible for her own resilience. Sociological stress research has shown that “differential exposure to stressful experiences is a primary way that gender, racial-ethnic, marital status, and social class inequalities in physical and mental health are produced,” that “minority group members are additionally harmed by discrimination stress,” and “stressors proliferate over the life course and across generations, widening health gaps between advantaged and disadvantaged group members” (Thoits 2010, p. 41). These findings do not only point to the fact that socio-economic factors may not be distant influential factors but also suggest that a one-sided individualistic perspective may not be adequate to deal with stress related mental problems. That is not to say that individual interventions to foster resilience are ineffective or unethical. Instead, we argue that if such interventions are not supplemented with policy interventions that take measures against structural social problems they might in the long run be only marginally effective and might even serve as handmaidens to those who want to block questions of (social) justice.
- Focusing exclusively on the individual level may not only hide questions of structural social problems and justice, rather the focus on the neurobiological mechanisms of resilience processes may also lead to misconceptions on the individual level. In Western industrialized countries there are high incidences of stress-related disorders that contribute more to the total all-cause morbidity than cardiovascular disease (Wittchen et al. 2011). Given the historically unprecedented levels of wealth and physical health, this might be puzzling at first sight, and it surely is a situation that demands more research on the concept of resilience and for fostering resilience. But an overly individualized neurobiological concept of resilience alone may not be the solution to this puzzle; on the contrary—following some sociological studies—it might even be counterproductive if applied exclusively: Alain Ehrenberg, who investigated the history of depression throughout the twentieth century claims that depression stems from an inadequacy of social context where success is attributed to, and expected of, the autonomous individual (Ehrenberg 2010a), insofar as depression in modern Western societies is a responsibility-related disease (Ehrenberg 2010b). Other authors point to the same direction (for example, Han 2010; Rosa 2013). Now if these claims are, at least partly, justified, delegating even more responsibility to the individual through resilience, regarding an individual’s own mental health—without addressing the structural factors that impact mental health—in the long run will not solve the problem of stress-related disorders. It becomes evident that underestimating the contribution of familial, social and cultural contexts by focusing just on the neurobiological underpinnings of individual resilience processes is actually a conceptual bottleneck. Due to the focus on individual neurobiological mechanisms (see for example, Egloff 2015; Freund and Staudinger 2015) only short- or mid-term functional resilience comes into sight where actual adversities are to be overcome. What is not reflected, though, is whether or not being more resilient is always something good, or put differently: if better is always something good. Empirical research suggests that being more resilient can also come with a price: Anthony (1987) in his longitudinal study found that resilient people also had exaggerated affect control, commitment phobia, and kept a great intellectual distance. These personal attributes might affect long-term (mental) well-being negatively but might not come into sight with a focus primarily on neurobiological mechanisms and their contribution to overcoming adversities situationally. Other empirical data point to the same direction: it seems that minimization of risk factors or aversive situations does not necessarily lead to well-being and absence of mental health problems, not more than developing resilience necessarily leads to subjective well-being (Goldstein and Brooks 2005; Lösel and Bender 1999; Luthar and Zelazo 2003; Masten and Wright 2010).

All in all, we suggest that a transdisciplinary view on resilience, on the level of experimental, clinical and theoretical research, that takes other empirical findings and conceptual criticism into account, makes it evident that resilience has to be thought of as a highly contextual (intrapersonal, interpersonal, social and cultural) concept (see for example, Fooken 2016). Following this insight, individual neurobiological mechanisms of resilience surely are an important Rosetta stone in the mosaic of the complex phenomenon of resilience. However, this perspective has to be complemented by approaches that stress the relational character of resilience processes on the one hand, and on the other, by those that capture not only (short-term) functional aspects but also involve perspectives of (reflexive) qualitative inner processes that do not necessarily lead to a quick adaption and functional resilience but arguably are important for long-term well-being and (authentic) personal development (for example, Garbarino 2008) differentiates between functional and existential resilience to stress these different perspectives).

Even though (non-neurobiological) concepts, which are closely interrelated with the notion of resilience, might very well irritate established views on health and disease, prevention, intervention and rehabilitation, it could be inspiring to further investigate concepts as the one of “salutogenesis” developed by A. Antonovsky (e.g., Antonovsky 1987). Similar to concepts of resilience, Antonovsky advances a focus on health instead of pathogenetic models and asks the question which factors are supportive of a person’s health in the face of stress. Central to the answer he provided on this question is what he calls “sense of coherence” (SOC). If this SOC is not robust enough, stressors will cause mental illness. This SOC has three components: comprehensibility, manageability, and meaningfulness. The crucial point here is that a sense of meaningfulness (that is, that things are valuable biographic elements) cannot be reduced to just the neurobiological underpinnings of short- or mid-term resilience and the maintenance of mental functioning. Arguably meaningfulness is a much broader category including aspects like life-history, family, interpersonal relations, society, history etc. To reduce the sense of leading a meaningful life to positive appraisal seems inappropriate and overly focused on individual appraisal and its neurological mechanisms alone.

## Epilogue: resilience as latent form of enhancement in transition

Since this paper is meant to discuss concepts of resilience in the light of their fruitful effectuation in a socially desirable and ethically acceptable manner, we will finally put the concept to another acid test. To argue that a one-sided conception of resilience is a form of latent enhancement in transition might seem paradoxical at first sight. Enhancement is defined by its primary goal to achieve some kind of improvement (primarily of biological resources) beyond what counts as normal, functional or even healthy (despite the lack of a consensual definition of health). In contrast, measures to improve resilience are understood as preventive measures protecting an individual’s social functionality and health. Notwithstanding the fact that it might be difficult to differentiate terminologically between enhancement and prevention (for example, Juengst 1997), it seems intuitively clear that the prevention of disease keeps in touch with a notion of health, and enhancement is something beyond that mainly hinging on concepts of social desirability.

Ultimately, if the expansion of the explanatory reach of the concepts of resilience as discussed above is taken into account, and if the implicit role of the concepts of normality and processes of normalization are accepted to be an inherent element of resilience which is then also designed in an overly individualistic way, we easily see how resilience might reflect some aspects of the enhancement debate which were criticized within the cultural, social and ethical discourse on enhancement practices:

1. Enhancement, too, concentrates on individual (biological) resources and usually does not incorporate social circumstances in its framework. Ideas of human enhancement argue for an adaptation of individual abilities to what is required to meet social or individual demands.^1^ Therefore, from a philosophical or ethical point of view, there is the charge of complicity which stems from adaptation to unmoral collective practices or circumstances and perpetuating these circumstances by that (on complicity see Singer 2011, p. 191ff).
2. Some critics also argue that normalization goes along with some enhancement practices, as we argued previously regarding the approaches in resilience research (for example, Scully and Rehmann-Sutter 2004).
3. Furthermore, enhancement also seems to be a concept that is hard to be confined. If better is always good, there seems to be no boundaries in sight. Even proponents of the enhancement practices struggle with this fact (for example, Agar 2010). The potentially open boundaries of the concepts of resilience seem to point in the same direction.

These similarities point to the fact that resilience, if understood in a one-sided, individualistic and reductionist mode can be interpreted as a latent form of enhancement in transition. It is a latent phenomenon because the pre-conditions for the transition from resilience to enhancement seem to be in place, however, a clear shift from resilience research into this direction cannot yet be diagnosed. It is a phenomenon in transition because we just start to see how the individualistic and more reductionist views of resilience, mainly grouped around neurobiological findings, begin to dominate the heterogeneous and sometimes confused landscapes of explanations, models and practices in the field of resilience. While it has to be stressed that a one-sided idea of resilience advocates the individual adaptation of capabilities to meet external demands without regarding the change in quality and scope of the respective demands (as does enhancement), there might still be a chance that the view on social circumstances, practices and institutions can safeguard resilience from venturing into the realm of enhancement.

It seems plausible to assume a general societal dynamic—at least in our post-industrial, Western societies—which requires individuals to prepare for (partly yet unknown) risks and demands and to preventively strengthen their resilience in unspecific ways which can hardly be differentiated from enhancement practices. This holds especially if resilience interventions move beyond the behavioral level to pharmaceutical interventions. Empirical data, that might be at first sight only illustrative, support this claim: The non-medical use of prescription drugs for pharmacological neuro-enhancement (understood as the use of these substances without medical indication to improve cognitive performance or mood) is more prevalent in groups who subjectively perceive their work-place and work-situation as very demanding (see Schröder et al. 2015; Sattler and Diewald 2015). Other studies showed that the use of pharmacological neuroenhancement seems to be more common in those who are less resilient to stress. One relevant reason for the use of these psychopharmalogical enhancement drugs is to better cope with stressful situations (Bagusat et al. 2018). This, however, is a general characteristic of competitive working environments in the twenty-first century. But these ever more competitive and demanding circumstances also seem to have consequences on the development of the personalities of young people. A recent study showed that perfectionism, understood “as a combination of excessively high personal standards and overly critical self-evaluations,” is increasing since the beginning of the 1980s among young people (Curran et al. 2017, p. 17). Especially self-orientated perfectionism and—more importantly here—socially prescribed perfectionism is reported. The authors of the study link these changes in personality to tougher social and economic conditions that young people face nowadays. Perfectionism in turn is a core vulnerability to a variety of disorders, symptoms, and syndromes (Flett and Hewitt 2002). Just strengthening an individually understood resilience might not be a solution for this vicious circle. And if this strengthening of resilience is done by psychopharmacology—like some authors suggest (see Wu et al. 2013) then the arguably fine line between fostering resilience and enhancement might get blurred. In this regard, it seems high time to take a closer philosophical and ethical look at the concepts of resilience and open a transdisciplinary debate about concepts, models and explanations of the resilience research on the one hand, and on practices to maintain or foster resilience on the other. Such a debate would prevent a one-sided, over-individualistic and reductionist view of resilience which will further contribute to a loose sight of the ever important fact that humans define and understand themselves as humans in relation to the meaningful lives of others.

## Footnotes

1 This version of enhancement is argued for by for example Bostrom/Sandberg 2008; for a critique see for example, Sandel (2009).
